# From exercise-associated lactate to the tubulin code: α-tubulin lactylation as a candidate mechanism for poststroke neural remodeling

**DOI:** 10.3389/fphys.2026.1972705

**Published:** 2026-10-28

**Authors:** Jing Guo, Hongxiao Wang

**Affiliations:** School of Physical Education, Hanshan Normal University, Chaozhou, China

**Keywords:** exercise, ischemic stroke, lactate, lactylation, microtubule dynamics, neuroplasticity, α-tubulin

## Abstract

Aerobic exercise promotes functional recovery after ischemic stroke, but the proposed link between an exercise-associated lactate pulse and durable neural remodeling remains unproven. The metabolite relevant to the hypothesis is L-lactate generated by skeletal-muscle glycolysis. In the principal mouse study, moderate treadmill exercise begun on poststroke day 6 (10 m/min, 30 min/day for 14 days) increased circulating and brain lactate together with α-tubulin lysine 40 (K40) lactylation and recovery; this regimen cannot be assumed feasible in patients with acute or severe disability. Electrically evoked skeletal-muscle contraction offers a preclinical exercise-mimetic option for nonambulatory subjects and increased circulating lactate in stroke mice, but it has not established a therapeutic lactate target, brain K40 lactylation, or clinical efficacy. Mechanistically, concurrent changes in L-lactate, K40 lactylation, and recovery show association rather than causal mediation. Exogenous L-lactate and dichloroacetate broadly alter metabolism and signaling, while substrate oxidation, HCAR1/VEGF signaling, BDNF/NMDAR-related plasticity, and lactylation of other proteins remain parallel explanations. The *in vivo* role of K40 lactylation has not been tested by a selective blockade. Moreover, the LacAVC phase IIa intravenous lactate trial was terminated after 28 enrollments for recruitment and protocol-related reasons. Although no results have been posted in the trial registry, a 2026 conference abstract reported preliminary safety and cerebral-bioavailability findings without establishing clinical efficacy or α-tubulin K40 target engagement.

## 1 Introduction

Recovery after ischemic stroke depends on limiting tissue injury and reorganizing surviving neural networks (Carmichael, 2006; Murphy and Corbett, 2009). Aerobic exercise can facilitate this plasticity; preclinical and translational studies report effects on neurotrophin signaling, synaptic function, dendritic remodeling, and functional recovery (Mang et al., 2013; Pin-Barre and Laurin, 2015; Ploughman et al., 2015; Xie et al., 2019). Yet the biochemical signal that couples a whole-body bout of exercise to cytoskeletal remodeling in injured neural circuits remains uncertain. This question is central to exercise physiology because a convincing mechanism must explain how an acute systemic perturbation is sensed by the brain and converted into a durable cellular adaptation.

Lactate is a plausible interorgan signal. It is continuously produced under aerobic conditions, becomes a major carbon shuttle as glycolytic flux rises, and functions as both an oxidative substrate and a signaling molecule (Brooks, 2018). During exercise, higher arterial lactate availability can drive net cerebral lactate uptake and oxidation (Quistorff et al., 2008; van Hall et al., 2009), while MCT1, MCT2, and MCT4 provide routes for lactate transport across the neurovascular unit and neuronal membranes (Bergersen, 2015). In experimental systems, lactate has been linked to exercise-responsive hippocampal BDNF signaling (El Hayek et al., 2019), HCAR1-dependent cerebrovascular adaptation (Morland et al., 2017), NMDA receptor-dependent plasticity-gene expression (Yang et al., 2014), and astrocyte-neuron lactate transfer required for long-term memory (Suzuki et al., 2011). These observations support the broader exercise-lactate-brain framework (Huang et al., 2021), but they do not identify a structural effector that directly links lactate exposure to neurite or axonal remodeling after stroke.

The discovery of lysine lactylation established a mechanism by which lactate metabolism can modify protein function (Zhang et al., 2019). More recently, α-tubulin lactylation was identified as a direct metabolic regulator of microtubule behavior (Sun et al., 2024). This Mini Review therefore evaluates α-tubulin K40 lactylation as one candidate metabolic-cytoskeletal branch through which exercise-associated L-lactate could contribute to structural neural remodeling after ischemic stroke. It does not assume that this branch is unique or dominant; throughout, we separate molecular plausibility, *in vivo* association, and causal mediation.

## 2 Skeletal-muscle L-lactate, practical exercise delivery, and brain exposure

For clarity, the metabolite proposed here is L-lactate, the stereoisomer produced from pyruvate by lactate dehydrogenase during skeletal-muscle glycolysis; D-lactate is not implicated. L-lactate formation is not restricted to hypoxia and rises when glycolytic flux exceeds local oxidative disposal, including during sufficiently intense aerobic work (Brooks, 2018). Human arteriovenous and isotope-tracer studies show that higher arterial L-lactate availability can increase cerebral uptake and oxidation during exercise (Quistorff et al., 2008; van Hall et al., 2009), while MCT1, MCT2, and MCT4 provide transport routes across the neurovascular unit and neuronal membranes (Bergersen, 2015). These observations establish systemic-to-brain transport physiology, not delivery of lactyl carbon to α-tubulin after stroke. In mice, acute exercise increased cerebellar MCT2 mRNA without a corresponding increase in protein, while pharmacological inhibition of cerebellar lactate transport impaired motor performance (Hoshino et al., 2016).

Experimental studies further show that L-lactate can reproduce selected neural responses to exercise. Monocarboxylate-transport blockade attenuated exercise-responsive hippocampal Bdnf activation, whereas systemic L-lactate engaged BDNF/TRKB signaling through a SIRT1-PGC-1α-FNDC5 pathway (El Hayek et al., 2019). L-lactate promotes adult hippocampal neurogenesis through MCT2 (Lev-Vachnish et al., 2019), and lactate-HCAR1 signaling contributes to cerebral VEGF expression and angiogenesis (Morland et al., 2017) as well as modulation of neuronal excitability (de Castro Abrantes et al., 2019). Lactate can also support oxidative metabolism and NMDAR-dependent plasticity-gene expression (Quistorff et al., 2008; van Hall et al., 2009; Suzuki et al., 2011; Yang et al., 2014). These routes may operate simultaneously and cannot be equated with α-tubulin lactylation.

The origin of the L-lactate pool relevant to tubulin lactylation remains uncertain. Ischemic tissue accumulates lactate, and astrocytes can supply locally generated lactate to neurons (Bergersen, 2015; Matsui et al., 2017). An exercise-associated rise in brain lactate or α-tubulin lactylation therefore does not demonstrate that the lactyl group originated in skeletal muscle. Here, “exercise-associated L-lactate” denotes the systemic pulse accompanying exercise, not a tracer-verified muscle-to-brain carbon flux. Stable-isotope tracing will be required to distinguish circulating L-lactate from ischemic or glial sources.

The exercise paradigm directly linked to α-tubulin K40 lactylation after stroke is preclinical: ischemic mice began treadmill running on postoperative day 6 at 10 m/min for 30 min/day over 14 days; starting on day 3 reduced survival (Zhang et al., 2026). This protocol is evidence for a delayed mouse rehabilitation model, not a prescription for patients with acute ischemic stroke. We do not propose running, swimming, or unsupervised high-intensity cycling during the acute phase. In medically stable patients able to participate, testable modalities include seated or recumbent cycling, body-weight-supported treadmill or overground walking, arm ergometry, and adapted aerobic-resistance circuits, with workload selected according to motor deficit and cardiopulmonary safety.

For patients who are bedridden or cannot produce purposeful limb movement, structured voluntary exercise may be impossible. Törteli et al. addressed this translational gap in mice: Electrically evoked skeletal-muscle contractions increased circulating lactate, attenuated neuroinflammation, and improved outcome after acute ischemic stroke; increased brain Mct1 expression was observed in stroke groups (Törteli et al., 2026). Neuromuscular electrical stimulation or functional electrical stimulation should therefore be described as a candidate exercise-mimetic method for generating a peripheral L-lactate signal without voluntary locomotion, not as established exercise therapy. The study was preclinical, did not test α-tubulin K40 lactylation, and does not demonstrate benefit in bedridden patients.

No human blood-L-lactate concentration or exercise intensity has been shown to induce α-tubulin K40 lactylation in the poststroke brain. A mechanistic clinical study would need to enroll medically stable patients, use individualized and closely monitored workloads, obtain serial lactate profiles, and prespecify stopping criteria; it should test whether a safe transient pulse occurs rather than require patients to reach an assumed therapeutic threshold. Acute exercise should not be initiated solely to generate lactate.

## 3 α-Tubulin lactylation adds a metabolic layer to the neuronal tubulin code

Microtubules are essential for neuronal polarity, long-range intracellular transport, growth-cone behavior, and structural remodeling of axons and dendrites. Their functional diversity is shaped by tubulin isotypes and post-translational modifications, collectively termed the tubulin code (Chakraborti et al., 2016; Magiera et al., 2018; Janke and Magiera, 2020). In neurons, relatively stable microtubules support long-distance transport, whereas locally dynamic microtubules permit neurite extension, branching, and growth-cone exploration. Manipulating α-tubulin acetylation can directly alter microtubule plus-end dynamics and axonal branching (Wei et al., 2018). Thus, a lactate-sensitive mechanism that tunes microtubule dynamics is structurally plausible in poststroke remodeling.

Sun et al. identified α-tubulin K40 lactylation and found that the modification is enriched in soluble tubulin dimers. They showed that intracellular lactate promotes α-tubulin lactylation, that HDAC6 can catalyze this reaction in a lactate-dependent manner, and that increased K40 lactylation enhances microtubule dynamics, neurite outgrowth, and branching in cultured hippocampal neurons (Sun et al., 2024). Mutation of K40 attenuated the structural effects associated with lactylation, providing site-specific mechanistic evidence. This work is particularly relevant to exercise physiology because it connects metabolite availability to a defined cytoskeletal substrate and a measurable neuronal structural response.

These findings also create an HDAC6 paradox. HDAC6 is a canonical cytosolic α-tubulin deacetylase (Hubbert et al., 2002), and changes in HDAC6 activity can alter microtubule dynamics and axonal growth (Zilberman et al., 2009; Tapia et al., 2010). The 2024 study, however, revealed an unexpected HDAC6 lactyltransferase activity that depends on its deacetylase catalytic machinery (Sun et al., 2024). In experimental stroke, ischemia caused abnormal nuclear redistribution of HDAC6, α-tubulin hyperacetylation, and microtubule overstabilization; HDAC6 dysfunction impaired dendritic maturation of adult-born neurons and reduced rehabilitation-associated recovery (Sheu et al., 2019). HDAC6 may therefore sit at the intersection of tubulin acetylation and lactate-sensitive K40 lactylation, but whether poststroke exercise engages this HDAC6-dependent switch remains unknown.

The proposed mechanism does not imply that more tubulin modification is uniformly beneficial. Neuronal microtubule stability must be tightly regulated, and tubulin post-translational modifications can shift both microtubule dynamics and function (Magiera et al., 2018; Janke and Magiera, 2020). For example, reduced α-tubulin acetylation produces microtubule hyperdynamics and axonal overbranching (Wei et al., 2018). K40 lactylation may therefore be beneficial only within specific spatial, temporal, and concentration ranges. It is better viewed as a metabolically responsive component of the tubulin code than as an intrinsically favorable modification.

## 4 Converging evidence in poststroke neural remodeling

Poststroke exercise produces structural and functional phenotypes consistent with, but not diagnostic of, a tubulin-based mechanism. Treadmill exercise after middle cerebral artery occlusion can increase dendritic and synaptic plasticity and improve neurological function (Xie et al., 2019), while the effects of exercise vary with timing, intensity, and rehabilitation context (Mang et al., 2013; Pin-Barre and Laurin, 2015; Li et al., 2020). The 2026 K40-lactylation study used only male mice and a delayed moderate treadmill regimen, and it reported poorer survival when exercise began on day 3 rather than day 6 (Zhang et al., 2026). These boundaries reinforce that the animal exposure cannot be translated into a universal acute-stroke exercise prescription.

Independent of exercise, exogenous L-lactate can be neuroprotective in experimental focal cerebral ischemia. L-lactate given after oxygen-glucose deprivation or transient middle cerebral artery occlusion reduced neuronal injury, and intracerebroventricular or intravenous administration produced benefits in rodent models (Berthet et al., 2009; Berthet et al., 2012). These studies predate protein lactylation and cannot distinguish substrate oxidation, redox effects, receptor signaling, protein modification, or their combination. They also do not establish that an intravenous exposure reproduces the spatial and temporal biology of an exercise-associated pulse.

Human evidence for exogenous L-lactate is unresolved. NCT04858139 was an exploratory randomized phase IIa study of a 20-min intravenous lactate infusion after endovascular treatment, designed primarily to test brain entry and metabolic effects. The registry lists the study as terminated after enrollment of 28 participants because recruitment was slow and a change in routine acute-stroke imaging and management risked selection bias; it does not report termination for a prespecified futility result, and no trial results have been posted in the ClinicalTrials.gov registry (ClinicalTrials.gov, 2025). A 2026 conference abstract reported preliminary safety and cerebral-bioavailability findings from LacAVC, including 11 lactate-treated and 10 placebo-treated patients. Lactate infusion increased the lactate-to-creatine ratio in healthy brain tissue, with no significant increase reported in the ischemic core or penumbra. These conference-level findings do not establish clinical efficacy or α-tubulin K40 target engagement (Salerno et al., 2026). The trial therefore does not establish that intravenous lactate is ineffective, but it also provides no evidence of therapeutic benefit. This incomplete clinical record is a substantial translational limitation and argues against recommending exogenous lactate.

The most direct convergence was reported in 2026. In ischemic mice, aerobic exercise increased circulating and brain lactate, global protein lactylation, and α-tubulin K40 lactylation together with neurogenesis, synaptic-plasticity markers, and functional recovery (Zhang et al., 2026). Exogenous sodium L-lactate produced a related phenotype, whereas dichloroacetate (DCA) lowered lactate-related readouts and attenuated exercise-associated benefits (Zhang et al., 2026). These interventions support involvement of lactate-related metabolism but are not specific perturbations of the K40 site. L-lactate can alter fuel supply, redox state, HCAR1 signaling, BDNF/NMDAR pathways, pH, sodium load, and many lactylation targets; DCA activates pyruvate dehydrogenase and broadly redistributes glucose, pyruvate, and lactate oxidation in neurons and astroglia (Itoh et al., 2003). Neither manipulation isolates α-tubulin lactylation.

Accordingly, concurrent increases in L-lactate, K40 lactylation, and recovery demonstrate association and temporal convergence, not causal mediation. K40 lactylation is detectable *in vivo* after exercise and L-lactate administration, but its functional role has not been established by selectively preventing this modification in the poststroke brain. HCAR1/VEGF-mediated angiogenesis, BDNF- and NMDAR-related plasticity, metabolic support, and histone or other non-histone lactylation remain viable parallel or interacting mechanisms (Suzuki et al., 2011; Yang et al., 2014; Morland et al., 2017; de Castro Abrantes et al., 2019; El Hayek et al., 2019; Lev-Vachnish et al., 2019; Zhang et al., 2019; Huang et al., 2021; Moreno-Yruela et al., 2022; Zu et al., 2022; Li et al., 2025; Sun et al., 2025; Zhou et al., 2025; Li et al., 2026; Qi et al., 2026). The current evidence cannot quantify how much, if any, of the exercise benefit is mediated specifically by α-tubulin K40 lactylation.

A stringent causal test requires more than lactate addition or DCA treatment. First, stable-isotope tracing should quantify the contribution of circulating exercise-associated L-lactate to neuronal lactate and tubulin-bound lactyl groups in peri-infarct tissue. Second, a temporally controlled, neuron-specific K40 perturbation should test necessity during exercise; because a K40 substitution may also abolish acetylation, the design requires acetylation-matched controls and rescue experiments before a lactylation-specific conclusion is possible. Third, factorial blockade of MCT transport, HCAR1, BDNF/NMDAR signaling, and candidate lactylation machinery should compare parallel routes rather than assume a single pathway. Fourth, direct measures of microtubule dynamics, axonal sprouting, and dendritic remodeling should establish the structural intermediate. **Figure 1** summarizes the candidate K40 branch and its evidentiary boundaries.

**Figure 1:**
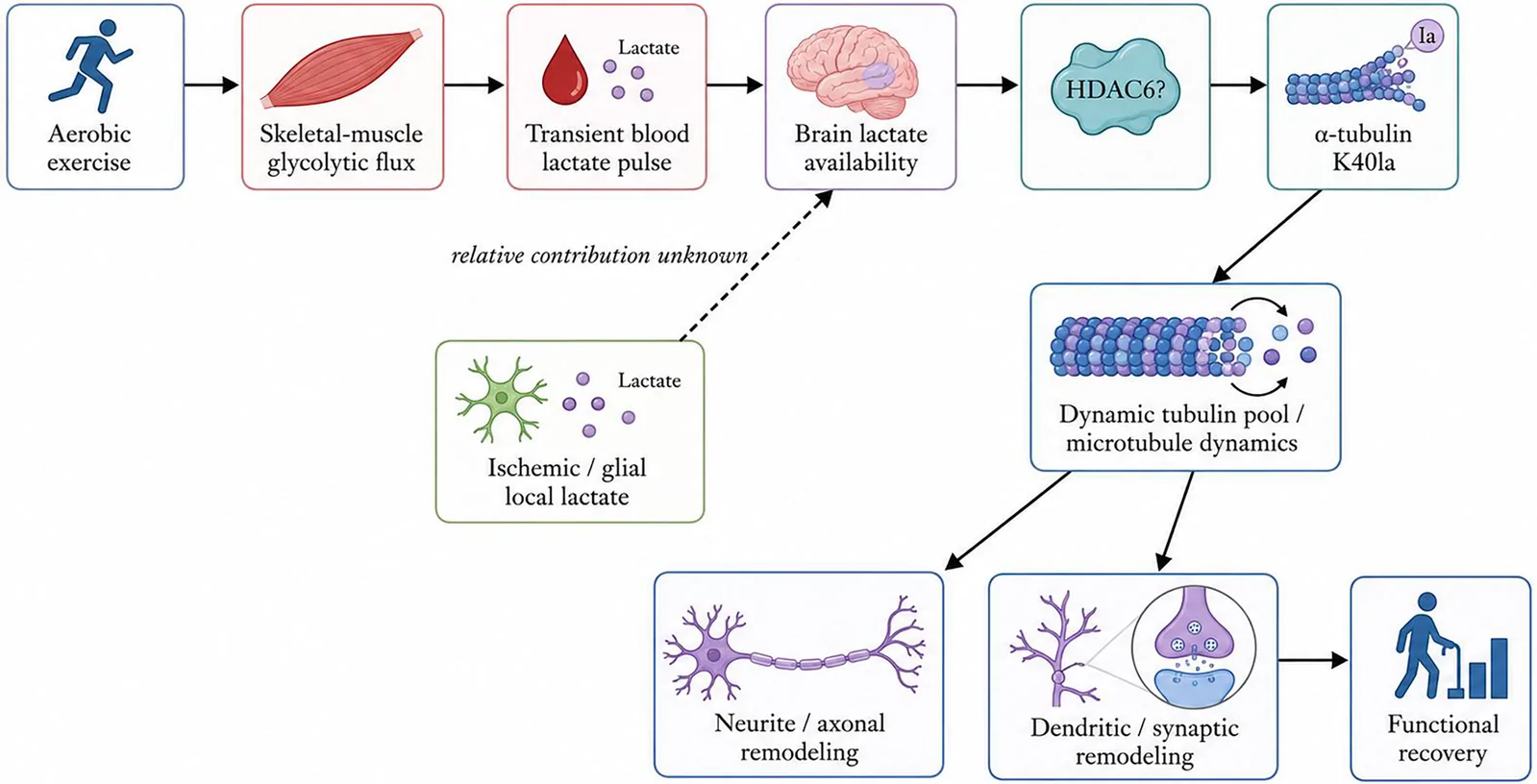
Candidate exercise -L-lactate -α-tubulin K40 lactylation pathway and evidentiary boundaries. The diagram illustrates a proposed metabolic -cytoskeletal pathway linking aerobic exercise to poststroke neural remodeling. Solid arrows indicate the direction of relationships within the schematic model, some of which have been demonstrated individually in experimental systems; they do not establish causal mediation of the complete pathway after stroke. The dashed arrow from ischemic or glial local lactate to brain lactate availability indicates an unresolved relative source contribution. The question mark beside HDAC6 indicates that its involvement in exercise-induced poststroke K40 lactylation has not been established. The complete sequence from exercise-associated lactate to functional recovery remains a mechanistic hypothesis. The aerobic-exercise illustration refers to the delayed experimental mouse paradigm and does not constitute an acute clinical exercise recommendation. K40la denotes lysine 40 lactylation.

## 5 Context-dependent lactylation: repair-associated signal or injury-associated modification?

It would be a major error to generalize from α-tubulin lactylation to lactylation as a whole. Lactylation is a regulated and reversible post-translational modification; class I HDACs and SIRT2 can remove lactyl groups from histone substrates (Moreno-Yruela et al., 2022; Zu et al., 2022). In ischemic stroke, biological effects vary with the modified protein, lysine site, cell type, and injury context. MeCP2 lactylation at K210/K249 reduces neuronal apoptosis and attenuates ischemic injury (Sun et al., 2025), whereas microglial H3K18 lactylation may promote an anti-inflammatory state through PLXNB2 (Li et al., 2025). Other substrates show the opposite phenotype.

By contrast, PLBD1 K155 lactylation increases PLBD1 abundance and exacerbates experimental ischemic brain injury (Zhou et al., 2025). In microglia, H4K5 lactylation drives an immunometabolic program characterized by increased glycolysis, reduced fatty-acid oxidation, lipid accumulation, and proinflammatory cytokine expression (Li et al., 2026). SOD2 K114 lactylation decreases SOD2 activity and is associated with oxidative stress, NCOA4-mediated ferritinophagy, and ferroptotic injury during cerebral ischemia-reperfusion (Qi et al., 2026). Thus, lactylation should not be treated as a unitary neuroprotective pathway. A more defensible model is that α-tubulin K40 lactylation represents one potentially repair-associated branch of a heterogeneous poststroke lactylome.

This heterogeneity makes L-lactate kinetics mechanistically important. A transient, well-cleared systemic pulse during exercise is physiologically distinct from sustained, localized accumulation in ischemic tissue. Kong et al. recently framed this distinction in Parkinson’s disease as a context-, compartment-, and time-dependent lactate problem, with exercise-like pulses separated from chronic maladaptive accumulation (Kong et al., 2026). That review provides a useful cross-disease kinetic framework, not direct evidence for stroke or for α-tubulin K40 lactylation. In stroke, concentration, exposure duration, transport, subcellular localization, and injury phase are likewise likely to determine which proteins are modified and whether the net effect favors repair or injury.

## 6 Discussion

Current evidence supports biological plausibility, not pathway-specific mediation. Skeletal-muscle glycolysis can generate a systemic L-lactate pulse, the human brain can import and oxidize circulating lactate (Quistorff et al., 2008; van Hall et al., 2009), intracellular lactate can support HDAC6-catalyzed α-tubulin K40 lactylation *in vitro* (Sun et al., 2024), and poststroke exercise in mice increases K40 lactylation together with repair and recovery (Zhang et al., 2026). These links form a coherent hypothesis. They do not establish that muscle-derived L-lactate reaches neuronal α-tubulin, that K40 lactylation is necessary *in vivo*, or that this route explains more of the exercise effect than metabolic, receptor, neurotrophin, vascular, or other lactylation pathways.

The evidence hierarchy should therefore be explicit. K40 lactylation has a site-specific molecular mechanism in cultured neurons (Sun et al., 2024); its abundance increases in poststroke brain tissue after exercise or exogenous L-lactate (Zhang et al., 2026); but its functional role *in vivo* remains untested. Lactate administration shows broad sufficiency for a related phenotype, and DCA shows sensitivity to broad metabolic manipulation, neither of which demonstrates K40-specific necessity. Even a K40 mutant would require careful controls because K40 is also acetylated. A convincing mediation claim will require cell-type-resolved target engagement, selective loss of the exercise benefit when K40 lactylation is prevented, restoration by a specific rescue, and direct microtubule readouts.

Clinical feasibility must be separated by recovery stage and disability. The delayed mouse treadmill protocol cannot justify acute running, swimming, or high-intensity cycling. Medically stable participants may be studied with seated or recumbent cycling, supported walking, arm ergometry, or adapted circuits; severely impaired or bedridden patients may require electrically evoked muscle contraction as an experimental exercise-mimetic approach (Törteli et al., 2026). In either group, serial blood L-lactate, hemodynamic and neurological monitoring, isotope tracing where feasible, peri-infarct target-engagement measures, and longitudinal outcomes are required. Because no therapeutic lactate threshold has been validated, intensity should be individualized for safety rather than prescribed to force a nominal lactate concentration.

NCT04858139 was terminated after 28 enrollments for recruitment and protocol-related reasons, and no trial results have been posted in the registry (ClinicalTrials.gov, 2025). Nevertheless, a 2026 conference abstract reported preliminary safety and cerebral-bioavailability data from 11 lactate-treated and 10 placebo-treated participants. Although intravenous lactate increased the lactate-to-creatine ratio in healthy brain tissue, no significant increase was reported in the ischemic core or penumbra (Salerno et al., 2026). These findings provide preliminary human bioavailability evidence but do not demonstrate clinical benefit or neuronal α-tubulin K40 lactylation. Exercise simultaneously changes cerebral perfusion, autonomic output, myokines, neurotrophins, substrate use, and vascular function (Mang et al., 2013; Pin-Barre and Laurin, 2015), while an infusion imposes different concentration-time, sodium, osmotic, and acid-base conditions. NCT04858139 was terminated after 28 participants for recruitment and protocol-related reasons and has not reported efficacy results (ClinicalTrials.gov, 2025). It therefore neither proves absence of benefit nor supports clinical use. Together with the nonspecificity of lactate and DCA manipulations, this record makes lactate supplementation and lactylation-mimetic therapy premature.

In summary, α-tubulin K40 lactylation is a plausible candidate link between exercise-associated L-lactate and cytoskeletal remodeling after stroke, but it is one branch within a pleiotropic lactate and exercise response. The current data establish molecular plausibility and *in vivo* co-occurrence, not K40-specific causal mediation. Defining a feasible stage-specific intervention, tracing lactate provenance, selectively testing K40 necessity with acetylation controls, comparing alternative pathways, and obtaining human target-engagement data are required before this mechanism can guide rehabilitation or therapy. **Table 1** summarizes the supportive evidence and the limitations that prevent a causal or clinical claim.

**Table 1:** Evidence for the candidate exercise-L-lactate-α-tubulin axis and the limits of causal inference.

Proposed link	Evidence context	Key finding	Interpretation/limitation	References
Skeletal-muscle glycolysis increases circulating L-lactate, and the human brain can take up and oxidize it.	Human exercise physiology and isotope-tracer studies.	Higher arterial L-lactate availability during exercise is associated with net cerebral uptake and oxidation.	Transport and oxidation are established, but the source of the lactyl group on poststroke neuronal tubulin is unknown.	(Quistorff et al., 2008; van Hall et al., 2009; Bergersen, 2015)
L-lactate can engage several brain plasticity pathways.	Healthy mouse, vascular, and neuronal models.	Metabolic support, BDNF/NMDAR signaling, HCAR1-VEGF angiogenesis, and neurogenesis are lactate responsive.	These parallel mechanisms may cooperate with or explain outcomes attributed to K40 lactylation.	(Suzuki et al., 2011; Yang et al., 2014; Morland et al., 2017; de Castro Abrantes et al., 2019; El Hayek et al., 2019; Lev-Vachnish et al., 2019; Huang et al., 2021)
Intracellular lactate can induce α-tubulin K40 lactylation.	Cultured neurons and biochemical assays.	HDAC6 catalyzes lactate-sensitive K40 lactylation of α-tubulin.	This provides a strong site-specific molecular mechanism.	(Sun et al., 2024)
α-Tubulin K40 lactylation can modify microtubule dynamics and neuronal morphology.	Cultured hippocampal neurons.	K40 lactylation increases microtubule dynamics and promotes neurite outgrowth and branching.	The mechanism is strong *in vitro* but has not been shown to be necessary after stroke.	(Sun et al., 2024)
HDAC6-dependent tubulin regulation contributes to poststroke neuronal maturation.	Experimental ischemic stroke, rehabilitation, and neuronal studies.	HDAC6 dysfunction is associated with α-tubulin hyperacetylation, altered microtubule stability, impaired dendritic maturation, and reduced rehabilitation benefit.	These studies provide a mechanistic bridge, but they did not test α-tubulin lactylation.	(Hubbert et al., 2002; Zilberman et al., 2009; Tapia et al., 2010; Sheu et al., 2019)
Aerobic exercise increases α-tubulin K40 lactylation after experimental stroke.	Male mouse ischemic-stroke model; delayed treadmill training.	K40 lactylation, repair markers, and functional recovery increase concurrently.	K40 lactylation is present *in vivo*, but no selective blockade shows that it mediates recovery.	(Zhang et al., 2026)
Exogenous L-lactate and DCA alter K40 lactylation and recovery-related outcomes.	Experimental ischemic stroke.	L-lactate produces a related phenotype; DCA attenuates several exercise-associated effects.	Both perturb metabolism broadly; neither isolates α-tubulin K40 lactylation.	(Itoh et al., 2003; Zhang et al., 2026)
Electrically evoked skeletal-muscle contraction can generate a peripheral lactate signal without voluntary locomotion.	Acute ischemic-stroke mouse model.	Electrical muscle stimulation increased circulating lactate and was associated with less neuroinflammation and better outcome.	Relevant to bedbound patients only as a preclinical exercise-mimetic concept; no human efficacy or K40 target engagement was tested.	(Törteli et al., 2026)
Intravenous L-lactate has preliminary human safety and cerebral-bioavailability evidence, but has not demonstrated clinical efficacy after ischemic stroke.	Exploratory randomized phase IIa LacAVC trial (NCT04858139); ClinicalTrials.gov registry and 2026 conference abstract.	The trial was terminated after 28 enrollments. Preliminary conference findings from 11 lactate-treated and 10 placebo-treated participants showed increased lactate-to-creatine ratio in healthy brain tissue, but not significantly in the ischemic core or penumbra.	Preliminary evidence of feasibility and cerebral bioavailability does not establish clinical efficacy, delivery to ischemic tissue, or neuronal α-tubulin K40 target engagement. Registry results remain unposted.	(ClinicalTrials.gov, 2025; Salerno et al., 2026)
